# Optimizing the Utilization of Steel Slag in Cement-Stabilized Base Layers: Insights from Freeze–Thaw and Fatigue Testing

**DOI:** 10.3390/ma17112576

**Published:** 2024-05-27

**Authors:** Peng-Cheng Song, Guo-Xin Chen, Ying-Jie Chen

**Affiliations:** 1 College of Hydraulic and Civil Engineering, Xinjiang Agricultural University, Urumqi 830052, China; songpc1996@gmail.com; 2 College of Civil Engineering and Architecture, Jiaxing University, Jiaxing 314001, China

**Keywords:** cement-stabilized steel slag mixture, discrete element method simulation, freeze–thaw damage, fatigue life, coupled load–freeze–thaw cycle analysis

## Abstract

This paper presents a study on the mechanical properties of cement-stabilized steel-slag-based materials under freeze–thaw cycles for a highway project in Xinjiang. Using 3D scanning technology the specimen model conforming to the real steel slag shape was established. The objectives of the study are as follows: to explore the sensitivity between the macro- and micro-parameters of the specimen and to establish a non-linear regression equation; and to study the changes in mechanical properties of materials under freeze–thaw cycles, fatigue loading, and coupled freeze–thaw cycle–fatigue loading. The results show that there are three stages of compression damage of the specimen, namely, linear elasticity, peak plasticity, and post-peak decline. Maximum contact forces between cracks and particles occur mainly in the shear zone region within the specimen. The compression damage of the specimen is a mixed tensile–shear damage dominated by shear damage. When freeze–thaw cycles or fatigue loads are applied alone, the flexural strength and fatigue life of the specimens show a linear relationship of decline. The decrease in flexural modulus at low stress is divided into the following: a period of rapid decline, a relatively smooth period, and a period of fracture, with a tendency to change towards linear decay with increasing stress. In the case of freeze–thaw–fatigue coupling, the flexural modulus of the specimen decreases drastically by about 50% in the first 2 years, and then enters a period of steady decrease in flexural modulus in the 3rd–5th years.

## 1. Introduction

Steel slag is one of the by-products of the steelmaking process, and since the 21st century, China’s iron and steel production has ranked the first in the world [1]. Along with the massive production of steel, the accumulation of steel slag has become one of the problems that needs to be solved in steel mills. Investigation shows that Chinese steel enterprises generally use traditional steelmaking methods, and each ton of steel smelting will produce 100~150 kg of steel slag [2,3], but the actual utilization rate of steel slag is less than 20%; the continuous accumulation of steel slag is not only a waste of land resources, but is also prone to bring about serious environmental pollution and safety problems [4,5]. Steel slag has good physical and mechanical properties, including hardness, crushing value, and abrasive value. If used properly, steel slag can partially or completely replace aggregates in mixes, which not only alleviates the shortage of construction resources, but also reduces damage to the environment. In the United States, the utilization rate of steel slag in road construction is close to 50%; in Europe, it is 43%; in Japan, it is 32.4%; and in China, it is only 7.6% [6], as of 2021. The cumulative amount of steel slag stockpiled in China exceeds 1.8 billion tons [7]. Road infrastructure construction can achieve the large-scale low-cost use of steel slag; road engineering will be one of the important development directions for the future use of steel slag.

At present, various experts and scholars have conducted corresponding research on the application of steel slag in road engineering. This research mainly focuses on the following aspects: when steel slag is applied to the asphalt surface layer, steel slag is mostly used to replace part of the aggregate in the asphalt mixture, and the corresponding analyses are carried out in terms of pavement performance and engineering applications. Studies have shown that electric steel slag is completely wrapped in the asphalt slurry, showing almost the same mechanical and physical properties as the traditional natural aggregates [8,9], and there is no precipitation of hazardous substances [10], while the addition of steel slag can significantly improve the performance of asphalt mixtures, which can improve the mechanical properties of the mixture, such as durability [11], resistance to permanent deformation, and resistance to wet damage [12]. Pasetto et al. [13] found that steel slag improved the dynamic modulus and resistance to the permanent deformation of warm mortar through dynamic shear rheology tests. Alinezhad et al. [14] showed that the fatigue properties of Stone Matrix Asphalt were comparable to those of electric arc furnace steel slag when it was used as a part of the coarse aggregate of warm stone matrix asphalt. Wu et al. [15] observed the microscopic agglomerate composition and morphological characteristics of steel slag and found, through indoor tests, that the use of steel slag as an aggregate improves the high-temperature performance and resistance to low-temperature cracking of Stone Matrix Asphalt. Goli et al. [16] comprehensively analyzed the experimental studies related to steel slag asphalt mixes, and the results generally showed that the use of coarse steel slag aggregate in warm mix asphalt enhanced the ability of asphalt mixtures to resist moisture damage and permanent deformation, and had better fatigue performance. The functionality of asphalt pavement, such as de-icing and self-repairing, is also improved accordingly due to the incorporation of steel slag aggregate. Gao et al. [17] tested the microwave heating capacity of steel slag with different grain sizes and verified the possibility of de-icing steel slag asphalt mixture pavement by using microwaves, which can improve the safety of road traffic in winter. Sun [18] and Plan et al. [19] carried out studies on steel slag asphalt mixes by inductive heating, microwave heating, and other methods; they studied steel slag asphalt mixtures in damage repair tests, and concluded that the use of appropriate heating methods can enable steel slag asphalt mixtures to obtain better healing and repair capabilities, and the addition of steel slag can improve the load–displacement relationship of the entire mixture. Xie et al. [20] comparatively investigated the temperature properties of steel slag and dolomite, and pointed out that steel-slag-based asphalt mixtures are able to maintain higher temperatures, and thus have a longer transport distance.

When steel slag aggregate is used in road subgrade, steel slag is mostly used as an aggregate into the mix, and the performance of the mix is studied accordingly. Li et al. [21] evaluated the feasibility of steel slag used in road subgrade, and analyzed the interaction between different stabilizing materials and steel slag as well as the relationship between stabilizing material dosage and performance. Wang et al. [22] used the discrete element method to simulate the damage process of cement-stabilized steel slag subgrade, and the results showed that the energy inside the structure was absorbed and dissipated continuously, and the micro-cracks went through the four stages of emergence, expansion, convergence, and penetration, and finally the structure was completely destroyed. Liu et al. [23] investigated the feasibility of applying steel slag in the subgrade of roads, and mixed different dosages of steel slag into the concrete and tested the mechanical and durability properties, and the tests showed that steel slag was beneficial to enhance the strength and shrinkage performance of the concrete. Li et al. [24] replaced fly ash pretreated steel slag with different volume ratios of anytime aggregate to study the mechanical properties of cement-stabilized steel slag (CSS) crushed stone mixes; the tests showed that the addition of 18% of fly ash in steel slag can effectively reduce the expansion rate of the mixes, and the mixes have the best mechanical properties when the ratio of steel slag to crushed stone is 1:1. Li et al. [25] found that CSS mixes have better dry shrinkage and temperature shrinkage performance than the cement-stabilized crushed stone mixes. The current research on the application of steel slag in road engineering has achieved a large number of research results, but mostly focused on steel slag as an additive blended into the aggregate, and less on steel slag as a total aggregate applied to road engineering research; moreover, for seasonal freezing conditions, the road in the freezing and thawing cycle and fatigue load coupling effect is less studied in the research. A semi-rigid base layer has the advantages of good slab bulk, high stiffness, strong diffusion stress, and good economic benefits, and has been widely used in China. Nowadays, the semi-rigid base layer represented by cement-stabilized mix and the road surface structure represented by asphalt mix have become one of the typical high-grade pavement structures in China. The application of steel slag as a full aggregate in a semi-rigid base layer represented by cement stabilization not only saves resources and protects the environment, but also realizes the scientific use of steel slag.

The purpose of this paper is to use steel slag instead of gravel as the main material in the semi-rigid road base layer, to study the strength change of CSS under the freeze–thaw cycle, to analyze the flexural strength and fatigue life of the material under the freeze–thaw cycle combined with discrete element software, and to analyze the deterioration of the flexural modulus of the material under freeze–thaw–fatigue coupling. The main original innovations are as follows: (1) freeze–thaw cycle research on CSS with all steel slag aggregate, and designing the number of freeze–thaw cycles according to the local climatic conditions; (2) adopting 3D scanning technology to construct a discrete element numerical simulation model of CSS with the real aggregate shape, and calibrate the detailed parameters of the discrete element numerical model by using the UCS test of the material under the freeze–thaw cycle; (3) adopting simulated beam specimens for simulation of material flexural strength under different numbers of freeze–thaw cycles, and achieving the simulation of flexural modulus change under freeze–thaw cycle–fatigue loading coupling. This paper can provide a reference for the application of CSS mix in cold regions, so that it can be widely used in road construction. 

## 2. Experimental Procedures

### 2.1. Materials

Semi-rigid grass-roots project quality is directly affected by the performance of raw materials; the performance indicators of raw materials used in the project should meet the corresponding specification requirements. In this paper, the research material is CSS mix, with the experimental use of cementitious materials for ordinary silicate cement; the coarse and fine aggregates used are steel slag, according to the corresponding specifications for the raw materials of the technical indicators for testing. According to the requirements of Chinese test protocols [26,27], the physical and mechanical properties and chemical composition of steel slag were tested; the physical and mechanical properties of steel slag are shown in Table 1. The group has used X-ray Fluorescence Spectrometer (XRF) to test the chemical composition of steel slag in the previous period; the test results are shown in Table 2 [28]. The cement used in the experiment is P∙O 42.5 ordinary silicate cement, and the technical indexes were tested according to Chinese test protocols [29], and the results are shown in Table 3. The indexes of the steel slag and the cement used in the experiment meet the requirements of the corresponding protocol. After testing, the indexes of steel slag and cement used in this test meet the requirements of the corresponding regulations.

### 2.2. Design of Mix Ratio for CSS

**Figure 1:**
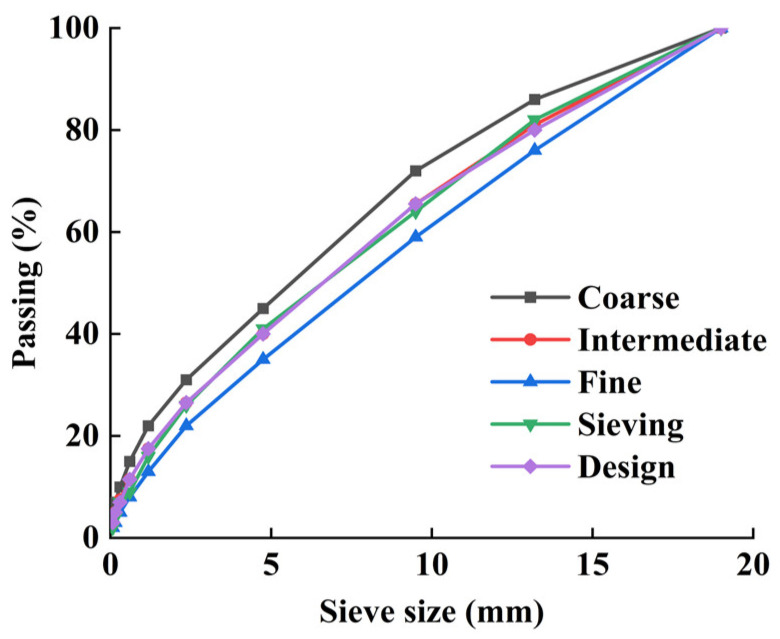
Gradation of CSS aggregates.

According to the Chinese specifications [30], the upper, middle, and lower limits of the grading range of steel slag aggregate are selected, the steel slag aggregate is sieved, and the mix gradation used in the experiments is selected after comprehensive consideration; the passage rate of the CSS aggregate with different sieve apertures is shown in Table 4, and the corresponding grading curves are shown in Figure 1. With reference to the specification [31], the three kinds of mixes with cement mass fractions of 4.5%, 5%, and 5.5%, which have been selected for grading, are subjected to compaction tests to determine the optimum moisture content and maximum dry density, as shown in Table 5.

### 2.3. Specimen Preparation

**Figure 2:**
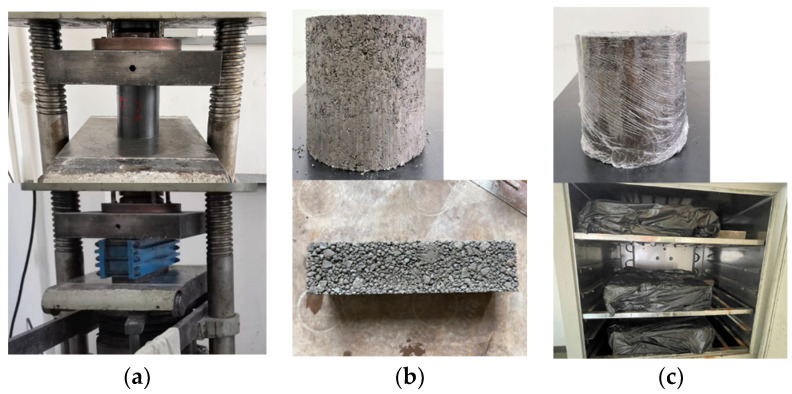
Specimen making: (**a**) mixture compaction; (**b**) specimen molding; and (**c**) specimen curing.

The specimens used in this study are cylindrical specimens and beam specimens of two kinds, cylindrical specimen size ϕ150 mm × 150 mm, and beam specimen size 100 mm × 100 mm × 400 mm, with specimens passing through the press to 1 mm/min compression speed compaction molding, with a compaction degree of 98%, and then placed in the temperature of 20 °C ± 2 °C, at humidity ≥ 95% of the environment in the standard maintenance; the specimen preparation process is shown in Figure 2. Cylindrical specimens need to be cured for 7 or 28 days depending on the test, and beam specimens need to be cured for 90 days. On the last day of curing, the specimen is removed and immersed in water for 24 h at room temperature, with the liquid level more than 2 cm above the top of the specimen.

### 2.4. Unconfined Compressive Strength Test

Perform the UCS test according to Chinese specifications (JTG E51-2009) [31]. Remove the cylindrical specimen after saturating it with water, wipe off the specimen surface with a dry towel, and observe the specimen surface; if there is obvious wear and tear, missing pieces should be discarded and supplemented with a new specimen. Place the specimen on the hydraulic pressure tester, set the loading rate of 1 mm/min, stop loading when the stress drops to 80% of the peak stress, and record the maximum stress during loading. The unconfined compressive strength (UCS) was calculated from Equation (1):

(1) $$R_{c}=P/A$$

where $R_{c}$ is the UCS (MPa); P is the maximum stress (kN); A is the specimen cross-sectional area (mm^2^).

**Figure 3:**
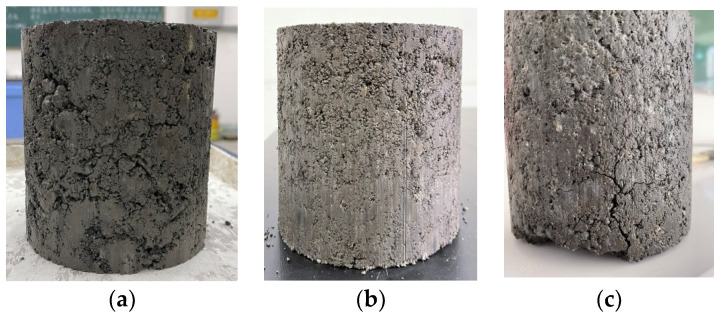
Cement-stabilized steel slag mix specimens with different cement dosages: (**a**) 4.5% cement dosage; (**b**) 5% cement dosage; (**c**) 5.5% cement dosage.

In order to determine the appropriate dosage of cement, different specimens were divided into three groups according to the cement dosages of 4.5%, 5%, 5.5%, with 9 specimens in each group; the measured 7-day UCS values of the specimens are shown in Table 6, and the 7-day curing specimens are shown in Figure 3.

By measuring the 7-day UCS values of the CSS mixtures with cement dosages of 4.5%, 5%, and 5.5%, respectively, it can be found that the compressive strength of the three kinds of cement dosage can meet the requirements of UCS (5–7 Mpa) in the Chinese specifications [30], and a comprehensive comparison of the CSS mixture specimens under the three kinds of cement dosage can show that, although the specimen strength can meet the requirements of specification when the cement dosage is 4.5%, the overall specimen is in a loose state, which will easily cause performance deterioration under the action of road traffic load and freeze–thaw cycle factors. Therefore, although 4.5% of the specimen strength can meet the specification requirements, the specimen as a whole is in a more loose state, and in the pavement under the action of driving loads and freeze–thaw cycle and other factors would be prone to performance deterioration, affecting the reliability of the road structure and service life. The strength of the specimen with 5.5% cement doping is much higher than the specification requirement, but the increase in cement doping makes the material as a whole prone to producing dry shrinkage cracks; and the content of calcium alumina produced internally is also increased, and calcium alumina and part of the steel slag with f-CaO have a certain micro-expansion effect [32], which will lead to part of the specimen inside the loading increasing in micro-cracks before loading, and under the action of the external load the stress will concentrate in the tip of the cracks, and after the ultimate load the energy-release crack growth occurs, so that the structural compressive strength and reliability are reduced, and there are more cracks on the surface of the specimen when it breaks. The limiting load is found after the energy-release crack growth, because the compressive strength and reliability of the structure is reduced, and the specimen becomes damaged as its surface cracks more. With the cement doping of 5%, the specimen as a whole is complete and compact, has a relatively smooth surface without obvious cracks, and its UCS meets the specification requirements, so this paper selects the 5% cement doping of CSS mixture specimen as a follow-up research object.

### 2.5. Freeze–Thaw Cycle Test

According to the Chinese specifications (JTGE51-2009) [31], the freeze–thaw cycle test was carried out on the specimens that reached the required curing time. The specific operation is as follows: on the last day of the maintenance period, the specimen is taken out of the standard maintenance room, grouped according to the number of freeze–thaw cycles, and immersed in water at room temperature, with the water surface higher than the specimen by at least 2.5 cm. At 24 h after immersion, the specimen is taken out of the water, and then placed in the low-temperature box of −18 °C for a freezing time of 16 h; in order to guarantee the freezing effect, the specimens should be spaced apart from each other by at least 2 cm. After the end of the freezing test, the specimen will be taken out, and then immediately placed into the 20 °C water for melting; the liquid surface should be higher than the specimen by at least 2 cm, and the melting time is 8 h. After reaching the predetermined melting time, the freeze–thaw cycle is finished, and the next freeze–thaw cycle is opened in the low-temperature box. After the last freezing test, take out the specimen and immediately put it into the 20 °C water immersion; the liquid surface is higher than the top of the specimen by at least 2.5 cm. After 24 h, take out the specimen, wipe off the water on the specimen’s surface, and for the UCS test, record the specimen’s destruction at the maximum pressure P (kN), and then calculate the UCS.

**Figure 4:**
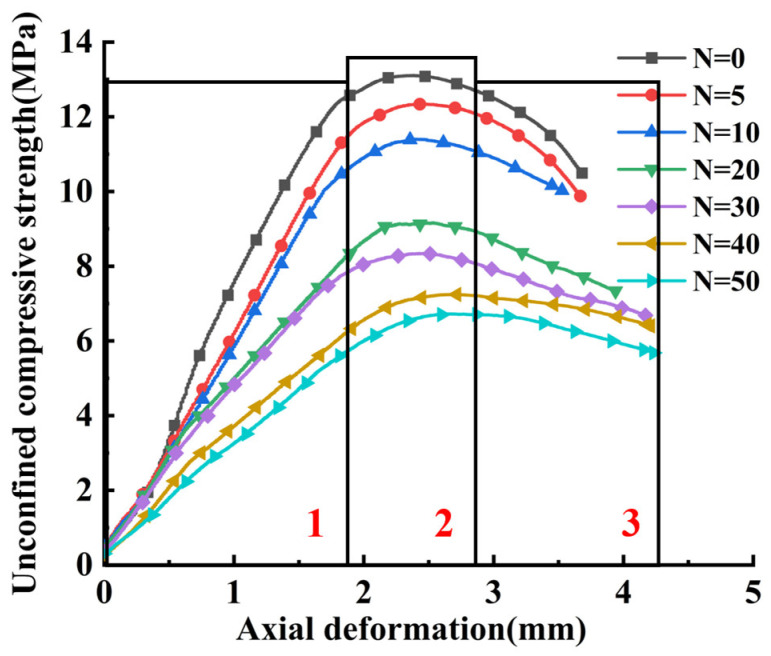
UCS–axial deformation curve of CSS under freeze–thaw cycles.

According to the relevant literature and meteorological data of Li et al. [33], and the equivalent indoor freezing and thawing number study of Wu [34], combined with the local climatic conditions in Xinjiang, the equivalent number of freezing and thawing cycles was selected as 10; the average annual number of freezing and thawing cycles in this region is about 100, i.e., the standard indoor freezing and thawing cycle of 10 can be approximated as the number of freezing and thawing cycles of about 100 in 1 year under the actual environmental conditions. Therefore, 5, 10, 20, 30, 40, and 50 times of indoor standard freeze–thaw cycles were chosen to simulate the number of freeze–thaw cycles in the overwintering period, the one-year period to the five-year period after the completion of the actual project. Cylindrical specimens were made, and the specimens were maintained for 28 days, and divided into 7 groups of 9 specimens each according to the number of freezing and thawing cycles, totaling 63 specimens for testing. The UCS–strain curves of the CSS mix specimens after 0, 5, 10, 20, 30, 40, and 50 freeze–thaw cycles are shown in Figure 4.

The changing patterns of the CSS UCS–axial deformation curves under different numbers of freeze–thaw cycles are roughly similar. They are as follows: (1) linear elasticity stage, (2) peak plasticity stage, and (3) post-peak decline stage. As the number of freeze–thaw cycles on the CSS increases, the peak compressive strength and the modulus of elasticity of the mix decrease, and the peak stress corresponds to an increase in axial deformation. This is because the internal fine structure of the CSS specimen changes after freeze–thaw cycle damage occurs, and the number of freeze–thaw cycles increases the size of the internal pores of the material and the scale of the gradual increase in the overall structure of the loosening, so that the material reaches the maximum stress of the axial deformation required to increase the elasticity of the specimen; at the same time, the aggregates contribute to the reduction in the strength of the cement, so that the compressive strength of the material reduces and the damage is constantly developing, and eventually affects the mechanical properties of the material.

### 2.6. Bending Strength Test and Fatigue Test

**Figure 5:**
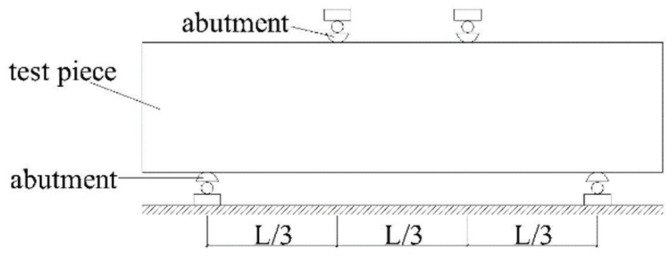
Schematic diagram of specimen loading.

The bending strength test and fatigue test both use the beam specimen; the loading mode schematic is shown in Figure 5, according to the Chinese specifications (JTGE51-2009) [31]. The beam specimen is loaded according to the three-point loading mode, with the span L = 300 mm, the loading rate is 50 mm/min, and the maximum load at the time of specimen damage is recorded; this load is the limiting load of the specimen at the time of damage, which is calculated according to Equation (2). Bending strength and continuous 10 Hz Havesine load waveforms are applied to beam specimens, as well as peak stress. In this paper, four stress ratios (K=σ/S) are selected to carry out the fatigue test, respectively, 0.6, 0.68, 0.76, 0.84. The number of times the load is applied at the time of fracture of the material is recorded, where K is the stress ratio, σ is the applied load for the fatigue test, and S is the limiting load at the time of damage of the specimen.

(2) $$R_{s}=P_{1}L/(hb^{2})$$

where $R_{s}$ is the bending strength (MPa); $P_{1}$ is the ultimate load at the time of specimen damage (N); L is the span (mm); b is the width of the specimen (mm); h is the height of the specimen (mm).

The flexural strength of the beam specimens with 0 cycles of freezing and thawing is shown in Table 7 with the fatigue life in Table 8.

## 3. Construction of Discrete Element Numerical Simulation Model Based on 3D Scanning Technology

### 3.1. 3D Scanning of Steel Slag Stones

The particle shape in PFC 3D 5.0 software is spherical by default, and the geometry of steel slag has an influence on the macroscopic mechanical response of CSS; in order to make the simulation model accurately reflect the mechanical response of the material, three-dimensional scanning technology and steel slag random throwing technology are used to construct a stochastic numerical simulation model of CSS.

In this study, a four-eye 3D scanner (GD-3dscanner) was used to scan the steel slag aggregate samples in three dimensions, The 3D scanner is produced by Mengyang Machinery Co., Ltd. in Wuxi, China. The main technical parameters of the three-dimensional scanner used in the test are as follows: measurement accuracy of 0.001~0.05 mm, a single scanning speed of about 1 to 3 s, a single scanning point in about 5,000,000, and there can be output stl, obj, and other formats of the file for the reverse-modeling software for post-processing, but also at the same time the program can find the measured object’s surface area, volume, and other quantities for the calculation. Because the steel slag is dark black overall, in the scanning process, the light absorption rate will be high due to insufficient reflectivity, resulting in large deviations in the machine’s 3D scanning, so in order to make the apparent shape of steel slag be accurately recognized by the machine, the surface of the steel slag should be uniformly sprayed with a developer before the scanning process, so as to increase its reflectivity, and to achieve the purpose of enhancing the accuracy of the apparent shape recognition. In the scanning process, in order to avoid the modeling deviation of the same object due to different viewpoints, the viewpoints should be selected as the geometric feature points with rotational invariance of the object to be measured, and the algorithm should be used to match the geometric feature points to achieve the multi-view point cloud splicing of the object to be scanned.

**Figure 6:**
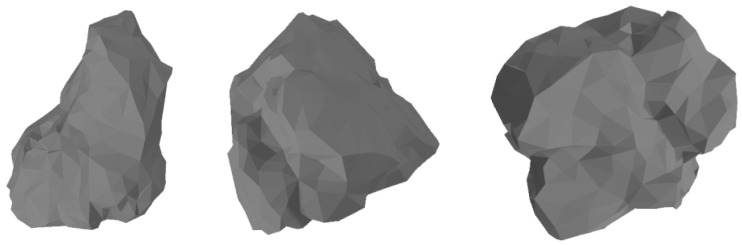
Typical steel slag scanning effect.

The point cloud data of steel slag stone obtained after scanning cannot be directly applied to the discrete element software, and it is necessary to carry out surface reconstruction of the point cloud data. In this paper, Geomagic Wrap 2021 software is used to process the scanned data. For the steel slag stone, the point cloud data processing steps are as follows: import the scanned file into Geomagic Wrap software, delete the steel slag stone noise points and redundant nails, and fill the holes with the function of the various surfaces to complement the integrity of the model to eliminate redundant point cloud data after the model is encapsulated in stl format for preservation. The processed steel slag three-dimensional model according to particle size is divided into three intervals (at this time, the particle size is not based on the actual particle size of the measured steel slag, but is equivalent to the equivalent particle size of the sphere volume), which were as follows: 4.75–9.5 mm, 9.5–13.2 mm, 13.2–19 mm. The different intervals were used establish a numerical simulation model of different particle sizes of steel slag stone. Figure 6 shows the scanning effect of different typical steel slag stones:

### 3.2. Construction of Stochastic Discrete Element Models Based on 3D Scanning

**Figure 7:**
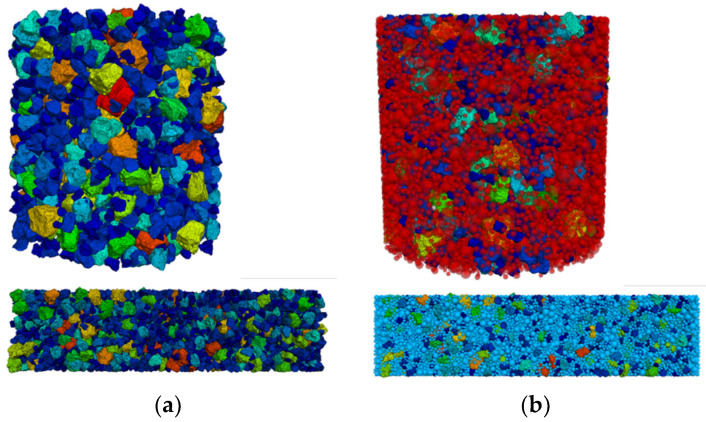
Discrete elemental modeling of specimens: (**a**) coarse aggregate steel slag cluster modeling; (**b**) modeling of cement-stabilized steel slag mixes.

Adopting the recommendations made by Ma et al. [35] in their study on coarse–fine aggregate thresholds and starting from the actual particle gradation, 4.75 mm was set as the coarse–fine aggregate threshold, with greater than 4.75 mm being set as steel slag stone, and less than 4.75 mm being set as steel slag sand. In order to simulate laboratory tests, the CSS model size and particle grading were set to be consistent with the indoor tests, i.e., ϕ150 mm × 150 mm for cylindrical specimen size and 100 mm × 100 mm × 400 mm for beam specimen size. Through the clump distribute bin command in PFC 3D software, it can be realized that the steel slag cluster model that has been imported into PFC 3D can be randomly fed into the specified program area according to the different particle size distributions and the designed gradation in Table 4, as shown in Figure 7a. In order to achieve the purpose of further improving the efficiency of computer operations, without affecting the calculation results, the steel slag sand can be simplified as a sphere ball in PFC 3D. The parallel bonding model was chosen for the inter-particle bonding model, and the final cylindrical model and the beam specimen model are shown in Figure 7b.

### 3.3. Sensitivity Analyses between Macro- and Fine-Scale Parameters

#### 3.3.1. Selection of Orthogonal Test Macro- and Fine-Scale Parameters

The modulus of elasticity E, peak strength $\sigma_{\mu }$, and Poisson’s ratio ν measured by uniaxial compression test can be used to represent the stress–strain pattern of UCS. Therefore, E, $\sigma_{\mu }$, and ν are chosen as the test indexes of the discrete element model in the orthogonal test. The contact model between the loading plate and the specimen at both ends is selected as a linear model, and the loading mode of lifting only the lower direction and fixing the upper plate is adopted, according to the research results of Cai et al. [36], and the speed is chosen to be 0.02 m/s considering the accuracy of the simulation test results and the operation efficiency of the simulation test computer, and the friction between the loading plate and the specimen is set to 0. In order to further improve the computational efficiency while ensuring the accuracy of the simulation test results, referring to Potyondy and Cundall’s suggestion [37], the effective modulus $\bar{E^{*}}$ and stiffness ratio $\bar{k^{*}}$ of the parallel bonded model take the same values as the effective modulus $E^{*}$ and stiffness ratio $k^{*}$ of the linear model, i.e., $\bar{E^{*}}$ = $E^{*}$ and $\bar{k^{*}}$ = $k^{*}$. The model has been simplified as described above, and the finalized fine-scale parameters to be assigned are as follows: parallel bond effective modulus $\bar{E^{*}}$, stiffness ratio $\bar{k^{*}}$, cemented tensile strength $\bar{\sigma_{c}}$, bond strength $\bar{c}$, angle of internal friction $\bar{\varphi }$, particle friction coefficient μ, normal critical damping ratio $\beta_{n}$, and bond radius scaling factor λ. For simplicity of calculation, the values of the fine-view parameters between steel slag coarse aggregates, between steel slag fine aggregates, and between steel slag coarse–fine aggregates were taken to be the same within each test.

#### 3.3.2. Orthogonal Test Design Scheme and Calculation Results

Sensitivity analyses between macro- and fine-scale parameters were conducted without considering the interactions between the levels of the factors. In this paper, five test levels were selected. With reference to the quantitative relationships between macro- and fine-scale parameters that have been identified in the literature on cement-stabilized mixes and concrete discrete elements [38,39], the values of the levels in the orthogonal test table were determined by analogical derivation, and the specific values are shown in Table 9.

For the eight test factors identified, orthogonal table $L_{50}(5^{11})$ was chosen to design the orthogonal table and orthogonal test, and the extra three columns were set up as empty columns, which were not listed in the following table because they were not involved in the calculation and occupied a large space. According to the orthogonal table, the modulus of elasticity E, peak strength $\sigma_{\mu }$, and Poisson’s ratio ν of each group of simulated specimens in uniaxial compression were obtained, and the orthogonal test scheme and corresponding experimental results are shown in Table 10.

#### 3.3.3. Sensitivity Analyses between Macro- and Fine-Scale Parameters

In order to distinguish the degree of influence of each factor on the measured test indicators, the level of significance was taken as α=0.05, and the high level of significance was chosen as α=0.01. A multi-factorial ANOVA was carried out to analyze the results of the test. Checking the F distribution table shows the following: $F_{0.05}(4,17)=2.96$, $F_{0.01}(4,17)=4.67$. When the statistic $F_{j}<F_{0.05}(4,17)$, it is considered that the statistic corresponds to a factor with insignificant influence and low sensitivity; when the statistic $F_{0.05}(4,17)\leq F_{j}\leq F_{0.01}(4,17)$, it is considered that the statistic corresponds to a factor with average significance and medium sensitivity on the measured test indicator; and when $F_{0.01}(4,17)<F_{j}$, it is considered that the statistic corresponds to a factor with highly significant influence on the measured test indicator and high sensitivity.

The orthogonal test data were processed by multi-factor ANOVA, and the results are shown in Table 11; at the same time, the data were grouped according to the test indicators, and the statistical sizes of the factors within the group were ranked in order of sensitivity, and the results are shown in Table 12.

**Figure 8:**
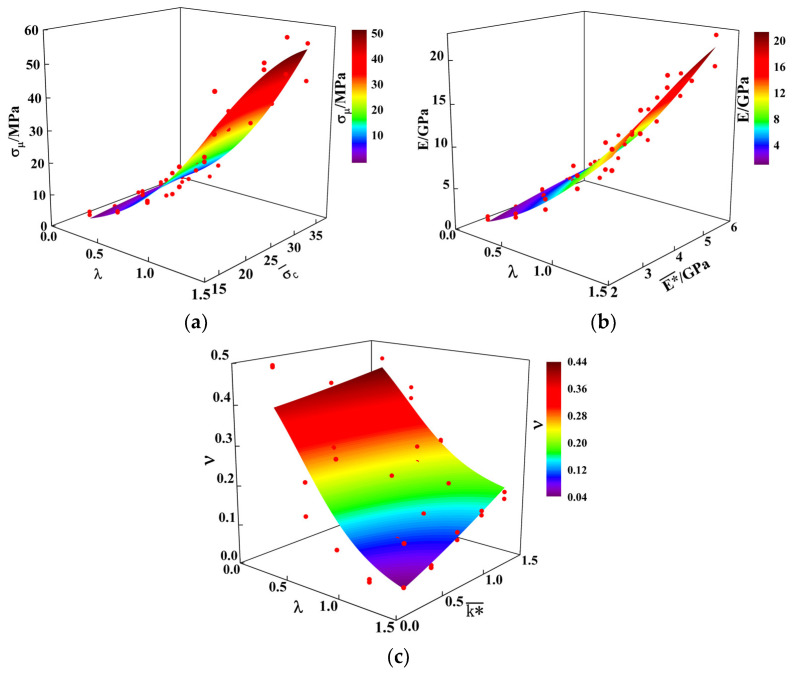
Interaction response surface of effective sensitive factors: (**a**) peak stress fitting surface; (**b**) elastic modulus fitting surface; (**c**) Poisson’s ratio fitting surface.

Select two experimental factors with high sensitivity within each indicator as effective sensitive factors for analysis. According to Table 12, the experimental factors selected for peak strength $\sigma_{\mu }$ are λ, $\bar{\sigma_{c}}$. The experimental factors selected for elastic modulus E are λ, $\bar{E^{*}}$. The experimental factors selected for Poisson’s ratio ν are λ, $\bar{k^{*}}$. To more intuitively express the degree to which each evaluation indicator is affected by various sensitivity factors, draw sensitivity analysis graphs for the following evaluation indicators and fit the response function, as shown in Figure 8.

As can be seen from Figure 8a,b, the peak stress $\sigma_{\mu }$ and elastic modulus E follow roughly the same trend when subjected to their respective high sensitivity influences, but the fitted surface for E is steeper, which suggests that E is more significantly influenced by the bond radius scaling factor λ, and the bond effective modulus $\bar{E^{*}}$. As λ increases, $\sigma_{\mu }$ and E gradually increase, while Poisson’s ratio ν gradually decreases. With the increase in stiffness ratio $\bar{k^{*}}$, the overall trend of ν is upward, and the increase in cemented tensile strength $\bar{\sigma_{c}}$ and $\bar{E^{*}}$ makes the corresponding $\sigma_{\mu }$ and E gradually rise.

With the comprehensive analyses of Table 11 and Table 12 and Figure 8, it can be seen that the elevation of λ makes the model $\sigma_{\mu }$ and E be elevated, while ν is reduced, indicating that the overall stiffness of the specimen is increased by λ elevation; the $\bar{\sigma_{c}}$ increase affects the bond strength of the bonding bonds between the material particles, resulting in a significant enhancement of the model $\sigma_{\mu }$; $\bar{E^{*}}$ leads to an increase in the hardening strength of the model and improves the local resistance of the material to elastic deformation, resulting in an increase in the modulus of elasticity of the model, whereas $\bar{k^{*}}$ mainly increases the transverse strain of the material when subjected to a force, decreases the stiffness of the material, and enhances its ν.

Using regression fitting of the test indicators with the fine-view parameters of the influencing factors with high sensitivity to them, the fit $R^{2}$ values are more than 0.86, the fit is good, and it can reflect the relationship between the fine-view parameters of the influencing factors with high sensitivity to them and the test indicators; see Equations (3)–(5).

(3) $$\sigma_{\mu }=-4.59+162.31\exp(-0.5{\left((\lambda -3.13)/1.21\right)}^{2}-0.5{\left((\bar{\sigma_{c}}-34.39)/24.33\right)}^{2})$$

(4) $$E=-2.94+60.79{\left(1+\exp(-((\lambda -1.44)/0.63))\right)}^{-1}*{\left(1+\exp(-((\bar{E^{*}}-2.13)/2.27))\right)}^{-1}$$

(5) $$\nu =0.56-0.7{\left(1+\exp(-((\lambda -0.46)/0.32))\right)}^{-1}*{\left(1+\exp(-((1.74-\bar{k^{*}})/1.22))\right)}^{-1}$$

where $\sigma_{\mu }$ is the peak stress in MPa; E is the modulus of elasticity in GPa; ν is the Poisson’s ratio; λ is the bond radius scaling factor; $\bar{\sigma_{c}}$ is the cemented tensile strength in MPa; $\bar{E^{*}}$ is the bond effective modulus in GPa; and $\bar{k^{*}}$ is the stiffness ratio.

### 3.4. Calibration of Discrete Element Parameters for CSS

In the discrete element software, the mechanical properties of the model are jointly determined by the type of inter-particle contact and the corresponding fine-scale parameters, and the inter-particle contact model is selected as a parallel bonding model. For the discrete element model calibration, referring to the relevant research of Xu [40], when the discrete element model is calibrated with the fine-view parameters, there are more types of contact models in the CSS discrete element model, and all types of contacts affect each other, so it is too difficult to calibrate directly. Therefore, a step-by-step calibration method is adopted for parameter calibration, as follows: (1) select the specimen to be calibrated for UCS testing and measure the physical and mechanical parameters of the specimen (E, ν, $\sigma_{\mu }$); (2) using Equations (3)–(5) and combining it with the test results, find the range of values of the fine-scale parameters which are the main factors influencing the macro-mechanical parameters of the test piece; (3) run the procedure to obtain the simulated UCS–strain curve, compare the simulated test with the loading–deformation curve of the indoor test, adjust the parameters when the difference between the two curves is more than 5%, and adjust the parameter with low sensitivity when the difference between the curves is less than 5% to make the curves closer. The calibration was carried out repeatedly in this way to obtain suitable calibration results. However, the UCS–deformation curve of the simulation test is not completely consistent with the real curve of the indoor test, especially in the post-peak softening stage, so we focus the control points of the simulation curve and the indoor loading–deformation curve on the online elasticity stage and the peak plasticity stage to achieve the similar slope and trend of the test curve and the simulation curve, with the same peak stress value of the curve and the similar peak point. At this point, we consider that the detailed parameters obtained from the calibration can be used for later modeling.

According to the coarse aggregate–coarse aggregate contact, coarse–fine aggregate, and fine aggregate–fine aggregate contact, the calibration parameters are shown in Table 13, Table 14 and Table 15, where the fine parameters corresponding to the number of freezing and thawing cycles in the table are the fine parameters between particles in the discrete element model built in the PFC 3D software when simulating the corresponding number of freezing and thawing cycles in the numerical simulation analyses.

**Figure 9:**
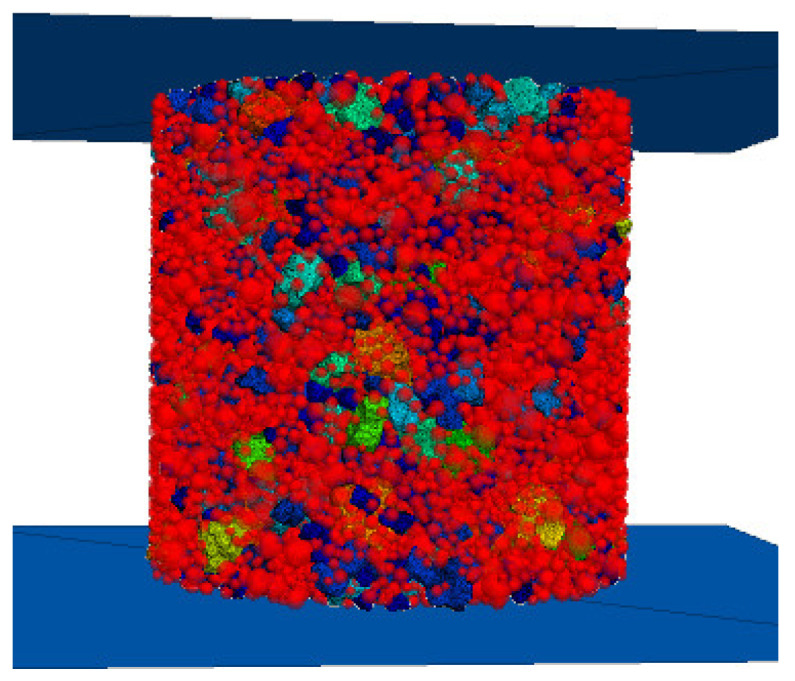
Simulation test of unconfined compressive strength.

**Figure 10:**
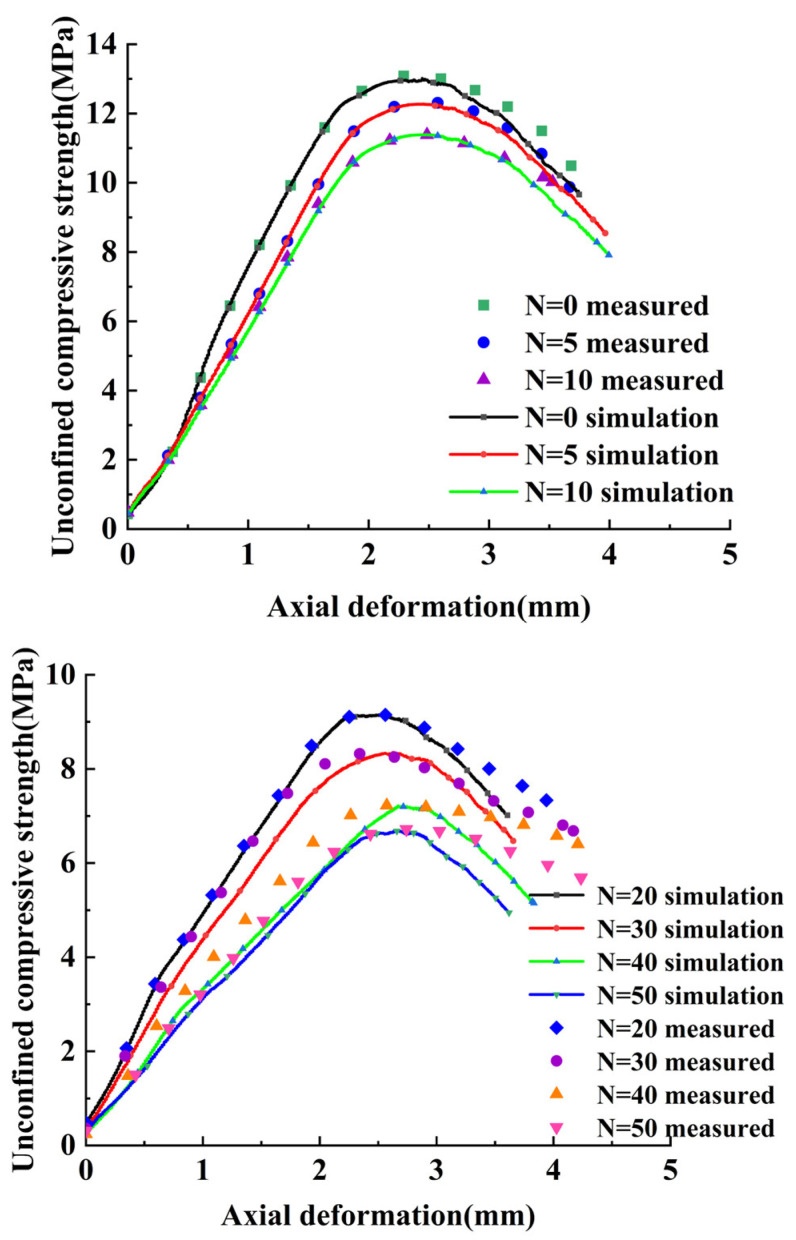
The test and simulation results.

The detailed parameters corresponding to different freeze–thaw cycles are selected in this section, and the numerical simulation of the corresponding freeze–thaw cycles is achieved by changing the detailed parameters of different groups in the model, and the uniaxial compression damage test is conducted to measure the UCS, and the loading mode is the same as the actual loading mode in the laboratory (see Figure 9), and the stress–strain curve of the numerical simulation model is plotted, and the results of the indoor tests are compared with the results in Figure 10, in which N is the number of freeze–thaw cycles. The stress–strain curve of the numerical simulation model was plotted and compared with the indoor test results, as shown in Figure 10, where N is the number of freeze–thaw cycles.

Comprehensive Figure 10 values and curve changes can be clearly known, with the numerical simulation tests in the CSS freeze–thaw damage curve in the ascending section and the UCS—axial deformation peak and the stress–strain curve of the test being generally consistent with the known stress–strain curve. However, the softening phase of the curve after the peak is affected by the fracture of some of the bonds between the particles, and the corresponding shear capacity of the material will decline; coupled with the fact that the particle shape of the numerical model is not diverse enough which leads to the occlusion between the particles being slightly smaller than that of the real test specimen, this leads to the numerical model of the softening of the peak being stronger than that of the laboratory measurement. The above results, by comparing the measured UCS–axial deformation curves under each freeze–thaw cycle number in the laboratory with the UCS–axial deformation curves obtained from the numerical simulation, show that the two are in good agreement in general. This also means that the discrete element model constructed in this paper is feasible in the simulation test to study the mechanical properties of CSS damaged by freezing and thawing.

## 4. Discrete Element Numerical Simulation Study of CSS

### 4.1. Uniaxial Compression Discrete Element Simulation of Freeze–Thaw Damaged CSS

**Figure 11:**
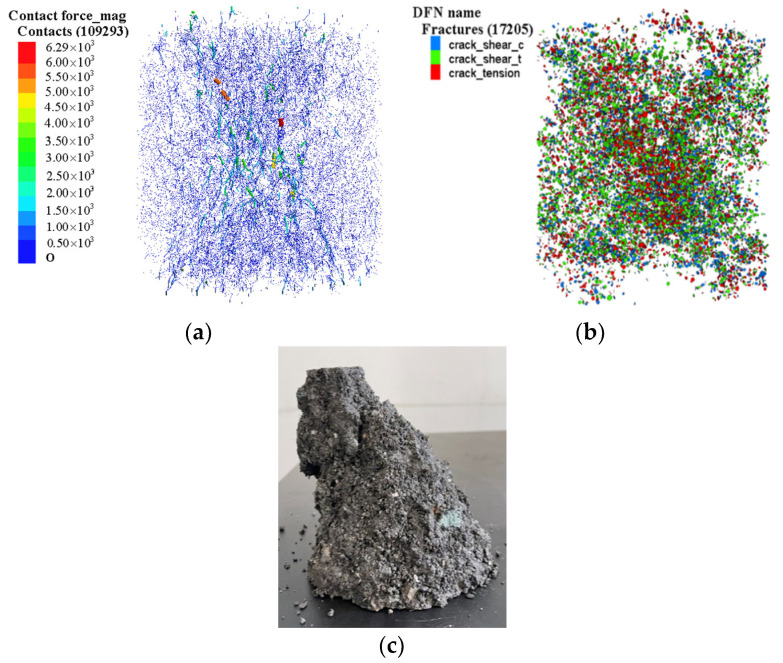
Macro-fine comparison of uniaxial compression damage of CSS specimens: (**a**) contact force distribution inside the specimen; (**b**) distribution of cracks inside the specimen; (**c**) specimen destruction diagram.

Uniaxial compression simulation experiments were carried out on the calibrated numerical model of CSS with different numbers of freeze–thaw times, and the contact force between particles and the development of cracks inside the model can be observed by the built-in commands of the PFC 3D software, and the corresponding analyses were carried out by combining the laboratory test results. Figure 11 shows the macroscopic and microscopic control diagram of the uniaxial compression damage of the specimen.

From Figure 11, it can be seen that the cracks are mainly concentrated in the 45-degree shear band, which is basically consistent with the location of the concentration of the maximum value of the contact force, and according to the maximum stress theory of Erdogan [41], the cracks start at the maximum stress in the circumferential direction of the particles. The crack sprouts from the tip at the maximum stress, combines with adjacent cracks and expands under compression, and the formation of micro-cracks leads to stress redistribution within the material, which in turn leads to the formation of shear bands during compression and specimen damage.

According to the information obtained from Figure 4 and Figure 10, the specific process of the uniaxial compression of cement-stabilized steel slag can be clearly divided into three stages based on three stage points. The first stage point shows that the stress of the specimen in the rising section of the curve is 0.7 times the peak stress, the second stage point shows that the stress of the specimen reaches its peak, and the third stage point shows that the test terminates. Select the first, second, and third stages to conduct in-depth exploration, and reflect the specific failure state of the water-stable steel slag mixture under uniaxial compression in the freeze–thaw environment by combining the changes in contact force between particles and crack development.

#### 4.1.1. Analysis of Contact Forces between Particles of CSS

**Figure 12:**
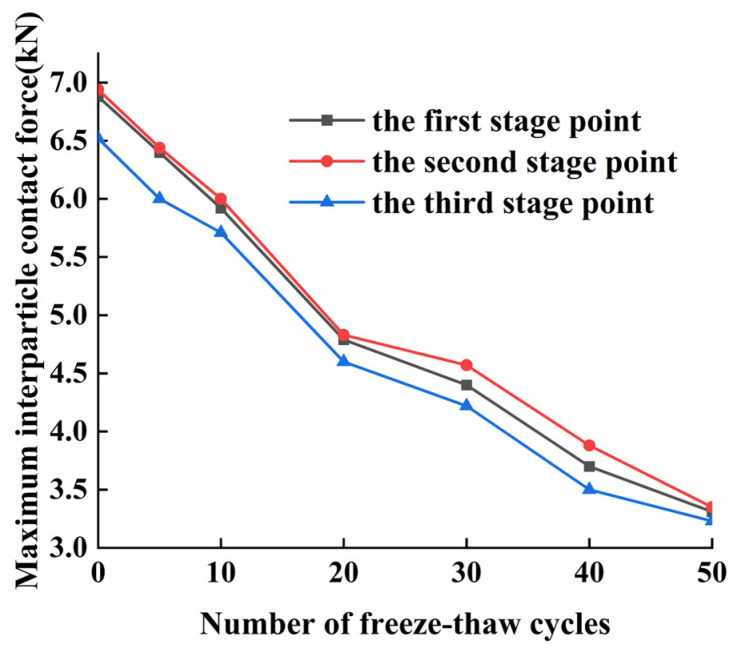
Maximum contact force of CSS damaged by freeze–thaw.

In the model, the contact force between particles is the force acting on the connecting bond between particles under the action of external loading, which produces relative motion and tendencies such as extrusion and overturning. According to the discrete element model, the contact force between the particles can be clearly reflected, and then can visually present the uniaxial compression of the CSS mixes with freeze–thaw damage. The peak contact force between particles of the CSS mixes at three different stages is analyzed under different numbers of freeze–thaw cycles, and the peak contact force at three different stages is visualized in Figure 12.

From Figure 11 and Figure 12, it can be seen that the maximum contact force inside the specimen mainly occurs in the 45° face region, and the contact force inside the specimen macroscopically corresponds to the internal shear stress on the material in general. With the increase in the number of freeze–thaw cycles, the maximum inter-particle contact force at each stage decreases gradually, in which the inter-particle contact force is the largest at the second stage, followed by the first stage, and the smallest at the third stage for the same number of freeze–thaw cycles. This is due to the fact that with the increase in the number of freeze–thaw cycles, the bonding force between particles in the model decreases and the occlusal force between particles is weakened, resulting in a gradual decline in the peak stress, which leads to a gradual decline in the peak contact force between particles. The first stage is the linear elasticity stage, in which the particles rely on the adhesive force and the inter-particle occlusal force to resist the external load; the second stage is the peak plasticity stage, at which time the internal particles of the material reach the maximum state of the contact force between the particles, some of the inter-particle stress exceeds the inter-particle bonding strength, the force chain ruptures to produce cracks, and at the same time the shear stress reaches the maximum stage. In the third stage, the external load continues to act, and the bearing capacity of the material decreases due to internal damage, which is manifested by the decrease in the stress and the maximum value of the contact force between the internal particles and the increase in the strain rate.

#### 4.1.2. Crack Development Pattern of CSS Model

**Figure 13:**
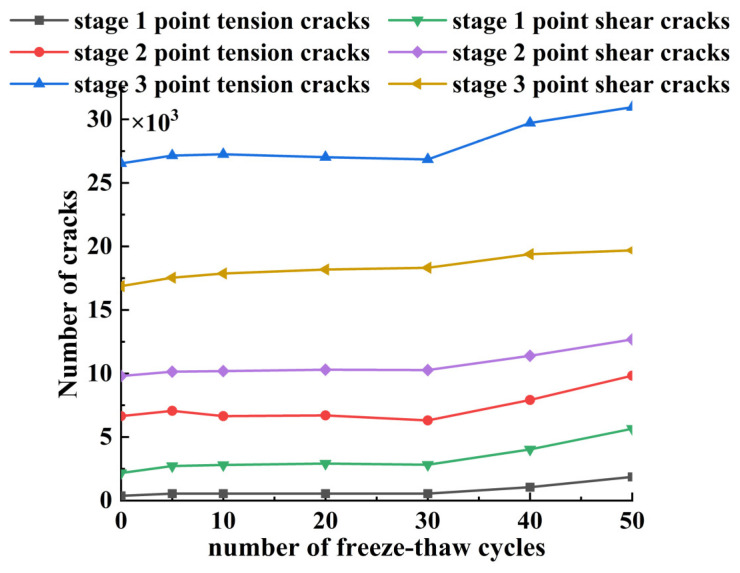
Number of cracks at three stages of freeze–thaw-damaged CSS (unit: pcs).

The parallel bonding model is the bonding model used in this paper for the contact between particles and particles of the material. When the parallel bonding strength is less than the stress on the parallel bond, the parallel bond will fracture, which can be defined as a micro-crack in the model of CSS; when the shear stress used for the bond is greater than the bonding shear strength, the crack formed is defined as a shear crack. Correspondingly, the cracks formed are defined as tensile cracks when the inter-particle tensile strength is not able to take up the stresses, and the connectivity and extension of the cracks can lead to macroscopic damage to the material. This section focuses on an in-depth analysis of the number of cracks generated in the uniaxial compression of CSS at two different stages of compression under freeze–thaw damage. Figure 13 shows the number of cracks generated at three different stages of the uniaxial compression of CSS.

According to the data presented in Figure 13, with the number of freezing and thawing cycles increased from 0 to 50, the number of microscopic cracks in the CSS model is in a continuous increase in the number of cracks, of which the number of cracks in the third stage point is significantly more than that in the second stage and more than that in the first stage. The first stage is the elasticity stage, when the material under pressure damage is minimal and produces the minimum overall number of cracks; the second stage is the peak stress point stage of the CSS material, when the specimen begins to damage, so the overall number of cracks rises; the third stage is the softening stage after the peak, when the specimen has produced serious damage, and the overall number of cracks increases sharply. At the initial stage, there are few or even no cracks, which is mainly due to the inter-particle friction and cementation forces at work. When the stress reaches the second stage, a significant increase in the number of cracks is observed; when the stress exceeds the peak value, the crack activity increases rapidly. In addition, the number of shear cracks is more than the number of tension cracks at the same stage, which indicates that the damage of the material under uniaxial compression is a mixed tension–shear damage with shear damage as the main damage.

### 4.2. Numerical Simulation of Frost–Thaw-Damaged CSS Mixes in Flexural and Fatigue Tests

#### 4.2.1. Bending Simulation Tests on Beam Specimens of CSS Mixes

**Figure 14:**
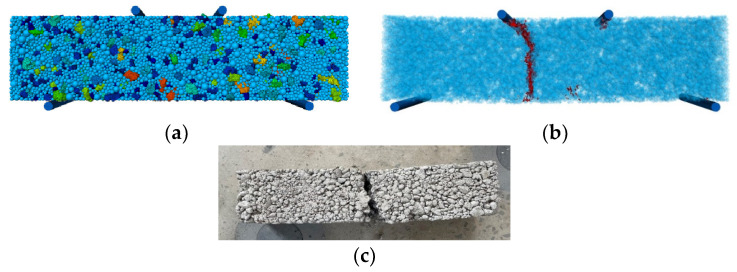
Simulation model of cement-stabilized steel slag bending strength test: (**a**) simulated specimen loading status; (**b**) simulated specimen fracture; (**c**) specimen fracture.

A three-dimensional discrete element beam specimen bending test model is established, the contact model between particles is selected as the parallel bond contact model, and the contact model between the model surface and the loading bar is selected as the linear model. The detailed parameters of the model are selected from Table 13, Table 14 and Table 15, and different groups represent different freeze–thaw cycles; the loading of the simulation model is carried out by the three-equivalent method of pressure, and the role of the loading axis is simulated through the establishment of a cylindrical wall on the specimen, and the lower wall is kept fixed throughout the loading process, and the upper wall is loaded with a loading speed of 50 mm/min. Specimen fracture is damage, and the specific cement stability bending model and damage diagram are shown in Figure 14.

The above calibrated fine-view parameters are used to simulate the freeze–thaw cycle 0, 5, 10, 20, 30, 40, 50 times of the beam specimen, to simulate the measurement of each model’s bending damage load, and to record the damage load of each model and calculate the bending strength according to the formula; in order to test the accuracy of the calibration of the fine-view parameters, the numerical simulation with freeze–thaw 0 times of the beam specimen bending limit load and the measured bending limit load of the indoor tests are recorded and the bending strength is calculated according to the formula as shown in Table 16.

**Figure 15:**
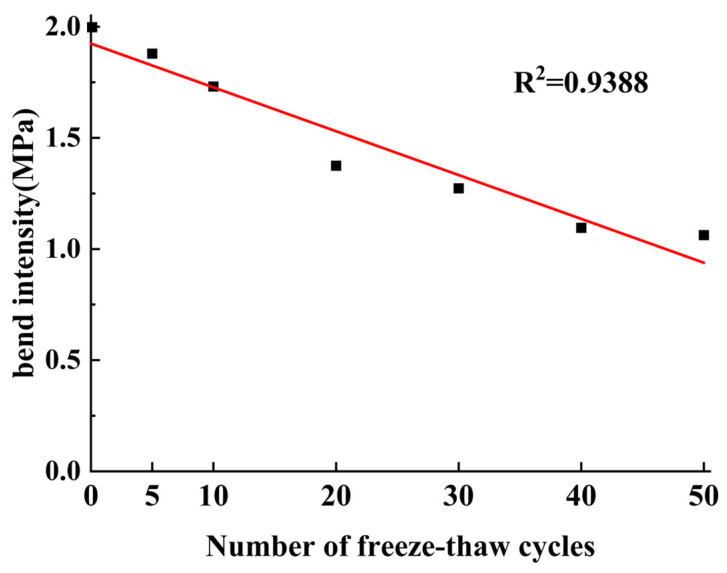
Relationship diagram between freeze–thaw cycles and bending strength.

As can be seen in Table 16, the measured ultimate damage load of the material in the laboratory is very similar to the damage obtained from the simulation tests, with an error of only 1.24%, which is in accordance with the test requirements. It can be considered that the fine-view parameters used for the above calibration is accurate, and can be used to simulate the bending strength of materials under different numbers of freezing and thawing cycles; after simulation, each group of values is shown in Table 17 below, and the data will have one-dimensional linear fitting, as shown in Figure 15 below, to obtain the relationship between the equation 6, R^2^ = 0.94, showing the fitting effect is good.

(6) y=−0.0197x+1.9239

where y is the bending strength, unit MPa; x is the number of freeze–thaw times

#### 4.2.2. Effect of Freeze–Thaw Cycles on Fatigue Life of CSS Mixes

**Figure 16:**
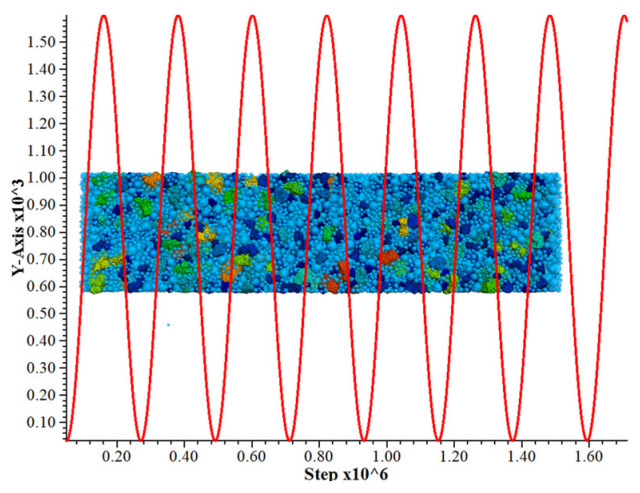
Schematic diagram of simulation model for cement-stabilized steel slag fatigue test.

In this section, the simulation is carried out in accordance with the indoor fatigue test, and the numerical model is chosen to have the same specimen size, particle gradation, loading position, and loading waveform as the indoor fatigue test, with the same loading method, and the number of fatigue loads when the material is fractured is recorded, and the simulation test is shown in Figure 16 below. The vertical coordinate in the figure is the applied load, and the horizontal coordinate is the running time step of the procedure.

**Figure 17:**
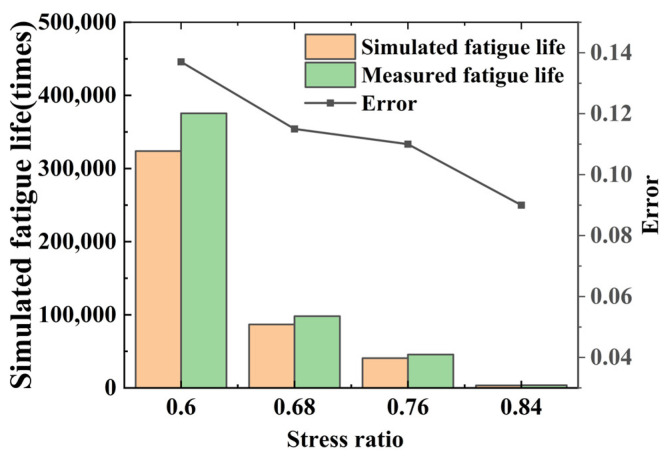
Comparison of simulated and measured fatigue life under different stress levels.

The calibrated fine-view parameters are used to simulate the middle beam specimens after 0, 5, 10, 20, 30, 40, and 50 times of freeze–thaw cycles, and the fatigue life simulation test is carried out for each simulated specimen, and the applied load stress level is the same as that of the indoor test, which is 0.6, 0.68, 0.76, 0.84, and the number of times subjected to cyclic loading at the time of damage of each model is recorded, as shown in the Table 18. In order to check the calibration accuracy of the fine-view parameters, the number of cyclic loads applied to the damage of the beam specimens in the numerical simulation model simulating freezing and thawing for 0 times was compared with the number of cyclic loads applied to the damage of the measured indoor tests, as shown in Figure 17.

As can be seen from Figure 17 and Table 18, there is still a certain gap between the simulated values of fatigue life and the measured values in the laboratory, but the overall trend is the same, with an error of about 11%; the error is lower at high stress levels, so it can be assumed that the fine-viewing parameter calibrated above can be used accurately, and it can be used to simulate the fatigue life of the material under different numbers of freezing and thawing times, as shown in Table 19.

Table 19 shows the fatigue life loss of the CSS specimens after different numbers of freeze–thaw cycles, and it can be seen that the freeze–thaw cycles have a very significant effect on the fatigue performance of the material. With the increase in the number of freeze–thaw cycles, the fatigue life of the material is greatly reduced; with the same number of freeze–thaw cycles, the larger the cyclic load on the material, the lower the fatigue life of the material.

### 4.3. Damage Modeling of CSS under Coupled Load–Freeze–Thaw Cycles

#### 4.3.1. Flexural Strength Decay Law of Materials under the Effect of Fatigue Damage

**Figure 18:**
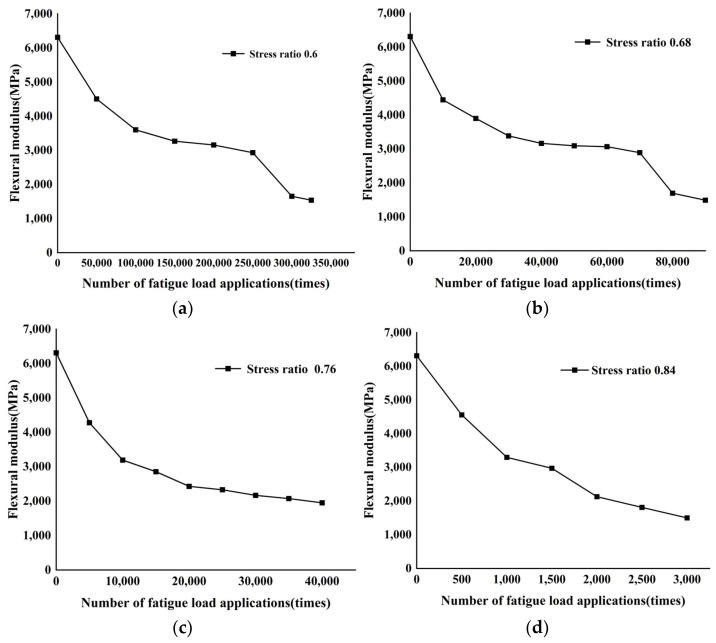
Relationship between bending modulus and number of loadings at different stress ratios: (**a**) stress ratios = 0.6; (**b**) stress ratios = 0.68; (**c**) stress ratios = 0.76; (**d**) stress ratios = 0.84.

In this section, based on the test data obtained from the fatigue simulation test of the CSS medium beam specimens in the previous period, the obtained data were equipartitioned, and the fatigue test was terminated at the equipartition point, and the bending simulation test was carried out to measure its bending strength, and then the change in bending modulus was obtained. The stress levels in the test were 0.6, 0.68, 0.76, 0.85, and the bending modulus decay law under different stress levels is shown in Figure 18.

As can be seen from Figure 18, in the simulation test with a stress ratio of 0.6, the bending modulus increases rapidly with the increase in the number of fatigue loads before 150,000 times, and the bending modulus decreases slowly and tends to flatten out between 150,000 and 300,000 times; after the fatigue load is applied for 250,000 times, the bending modulus appears to change abruptly and decreases rapidly, and at this time, the specimen fracture occurs. The whole process is divided into three phases, i.e., a period of rapid decrease in bending modulus, a period of relatively stable bending modulus, and a period of sudden change in bending modulus fracture. When the specimen is under the stress ratio of 0.68, it has the same three stages as the specimen with the stress ratio of 0.6, but the rapid decline of the bending modulus of the specimen with the stress ratio of 0.68 decreases faster than that of the specimen with the stress ratio of 0.6, and the specimen also ruptures after the steady period. In the stress ratio of 0.76, in the whole fatigue loading process, the bending modulus change is divided into two stages: the first stage of the bending modulus is the rapid decline stage, accounting for about the first 20% of the whole process; the second stage is a smooth decline stage, and the whole decline stage is almost a linear stage, with the material mainly in the range of the latter 80% in the actual service process. When the specimen is loaded with a stress ratio of 0.84, the bending modulus of the material decreases rapidly with the increase in the number of fatigue loadings, which is completely different from the trend of the bending load after fatigue loading at the other three stress levels. The reason is that when the specimen is loaded at a high stress level, the cumulative rate of internal damage is much higher than that of the other three, so the damage of the specimen is more obvious. By analyzing the above test results, it can be observed that under fatigue loading with different stress ratios, the specimen’s flexural modulus shows an obvious decreasing trend. The stages of decline are different but generally decrease with the increase in stress ratio; the sudden fracture period disappears after the increase in stress ratio, and each stage shows a rapid decline in the initial stage, and the decline slows down with the increase in the number of loading actions, and finally the specimen is destroyed. For the low-level stress specimens, a significant critical value of change in flexural modulus was observed in the tests.

#### 4.3.2. Bending Modulus Decay Law under Fatigue Loading–Freeze–Thaw Cycle Coupled Damage

In order to determine the number of applied fatigue loads, the whole fatigue loading process is equivalent to the actual loading process from the perspective of damage accumulation in damage mechanics, in which the initial state of the material is the starting point and the final fracture of the beam is the end point, and the year is taken as the fatigue loading unit to perform the equivalent analysis for the whole loading process. According to the actual field coring investigation and overhaul engineering experience, the semi-rigid grass-roots level occurs overall broken generally between 15 and 20 years (which can be considered as the 15 years of destruction group for when the traffic volume is larger, and the amount of annual fatigue action is greater; or it can be considered as the 20 years of destruction group for when the traffic volume is lighter, and the amount of annual fatigue action is smaller). Fatigue loads are classified according to light load fatigue and heavy load fatigue, and the number of fatigue loads applied per year for light load fatigue simulation is 1/20 of the fatigue value of the specimen. And heavy-duty fatigue specimen simulation of the number of times per year to apply fatigue load is 1/15; for the number of times per year as a unit, when applying fatigue load, apply the program to carry out the test, the simulation of the specific design is as follows:

According to the above, the equivalent number of freeze–thaw cycles in Xinjiang is 10 times per year; according to the conclusion of the fatigue load on the bending modulus damage test above, a low level of stress is chosen for the test coupling, i.e., a fatigue load with a stress ratio of 0.6 is chosen. A fatigue life of 323,825 cycles per year was obtained from the tests. The groups are grouped according to heavy load and light load, and the non- freeze–thaw group is set as the control group. The simulation unit settings are shown in Table 20.

**Figure 19:**
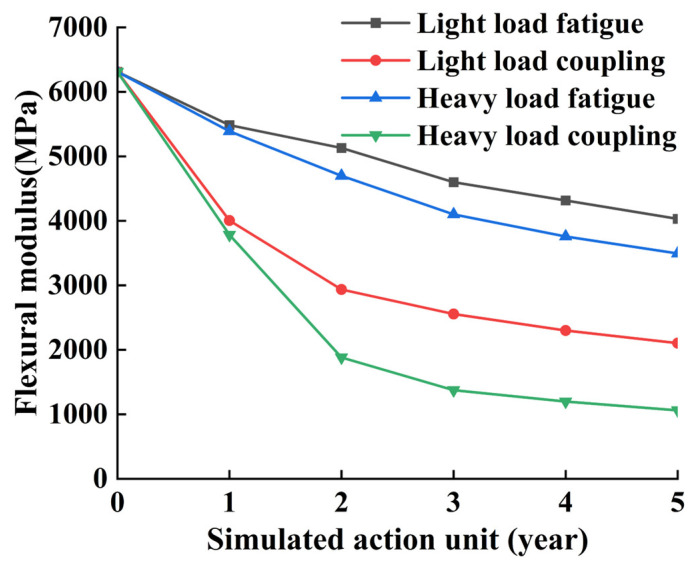
The attenuation law of bending modulus of cement-stabilized steel slag under different working conditions.

Based on the above scenario, different types of acting units were selected during the tests. The material was subjected to 5-year fatigue loading–freeze–thaw cycle coupled simulation tests to obtain the modulus decay law of CSS under different simulation combinations, and regression analyses were carried out, and the results of the tests are shown in Table 21 and Figure 19.

For CSS mixes, under the coupling of fatigue–freeze–thaw cycles, the flexural modulus decreases rapidly in the early stage, and then slows down in the later stage; the curve tends to flatten out, and the overall trend of the curve has the characteristics of first being fast and then slow. Heavy loads, due to their higher number of fatigue loads per year, show a larger decrease compared to light loads, but the trend of the curves does not change significantly. Under the same loading conditions, comparing fatigue action alone and freeze–thaw cycle–fatigue load coupling action, the coupling action caused the degree of damage to the specimen to increase substantially, especially the coupling action of the first two years. The bending modulus decline rate increased significantly, and the bending modulus as a whole showed a two-stage change in the freeze–thaw cycle, and fatigue loading under the dual action of the specimen in the first two years of the damage is extremely intense with a cumulative loss of the bending modulus for the original half of the original. After two years, the decrease in bending modulus enters into a stable period; at this time, the rule of change is basically consistent with the single fatigue loading. The overall decrease in the flexural modulus of the specimens under single fatigue loading was relatively smooth over a 5-year period. Regression analysis was performed on the simulated test set data and the results are shown in Table 22, where y represents the flexural modulus and x represents the number of years of the coupled simulation unit.

After dividing the number of fatigue loads into unit years according to different bearing degrees, coupling with freeze–thaw cycles, and fitting the test data, four different bending modulus regression equations were obtained. According to the results of the regression analysis in the above table, it can be seen that the regression equations are extremely complex, which can also reflect how the modulus attenuation of the material under the damage of the coupling effect is characterized by large variability. However, on the whole, the curves show two stages of decline, i.e., the rapid decline period at the beginning and the gentle decline period at the end.

## 5. Conclusions

In this paper, steel slag is used as a full aggregate in the cement-stabilized semi-rigid base layer to determine the optimum mix ratio of the material. The indoor freeze–thaw cycle test was conducted to study the strength deterioration pattern of the CSS mix and the change in the UCS–axial deformation curve. Combined with the discrete element program, the bending, fatigue simulation, and load–freeze–thaw cycle coupling simulation tests were carried out on the beam specimens of the CSS mix, and the main conclusions are as follows:

(1) The best cement dosage of the specimen is 5%; although 4.5%, 5%, and 5.5% are three kinds of cement dosage that can meet the specification of the extra heavy traffic state of the pavement grass-roots bearing capacity requirements, the cement dosage of 4.5% results in a specimen with an overall looseness of surface roughness, with the fine aggregates falling off; and cement dosage of 5.5% results in a specimen which, after 7 days of the curing period, due to the expansion of ettringite in the excess cement and the micro-expansion of the steel slag, had cracks appeared on a small part of its surface. The specimen with 5% of cement mixing had a better integrity and no obvious defects.
(2) Using three-dimensional scanning technology and orthogonal experimental design to analyze the sensitivity relationship between the parameters in the discrete element software, we obtained that λ has a significant effect on $\sigma_{\mu }$, E, and ν, and that $\bar{\sigma_{c}}$, $\bar{E^{*}}$, and $\bar{k^{*}}$ have a significant effect on $\sigma_{\mu }$, E, and ν, respectively, and we fitted the non-linear relationship equation between the parameters; after the uniaxial compression simulation, the obtained stress–strain curves have a high degree of restoration in comparison with the laboratory-measured curves, which indicates the correctness of the calibration parameters. The maximum value of the contact force and the main cracks are distributed in the shear zone region of the specimen, and the number of shear cracks throughout the compression damage is more than the number of tension cracks, so the compression damage is essentially a mixed tensile–shear damage with shear damage as the main damage.
(3) In the bending strength experiment, the error between the measured laboratory breaking load and the breaking load obtained from the simulation test is only 1.24%. The error between the results of the fatigue test and the simulation is 8–14%, which decreases when the level of load acting on the specimen is increased. The specimen bending modulus under the action of fatigue loading at relatively low stress has three stages of change: a rapid decline period, a relatively smooth period, and a sudden change fracture period. When stress ratio is small, there is a critical value of change; when the stress ratio increases the decline phase is reduced, and the sudden change fracture period disappeared; in the freezing and thawing cycle–fatigue loading coupling, the first two years of the bending and tensile modulus drastically decreased to the original 50% or so, and with 3–5 years of the bending and tensile modulus, the decline tends to level off.
(4) The next step could be to continue the study in the following two parts: research on the mechanical properties of specimens with different grades and cement contents subjected to freeze–thaw cycles; and research on the effect of multi-factor coupling (e.g., temperature–wet/dry-fatigue coupling) on the mechanical properties of cement-stabilized steel slag base layers.

## Figures and Tables

**Table 1:** Physical and mechanical properties of steel slag.

Serial Number	Test Items	Standard	Test Result
1	Crushing value/%	≤26	3.3
2	Needle-flake particle content/%	≤18	6.3
3	Dust content below 0.075 mm/%	≤2	1.2
4	Water absorption/%	≤3	1.4
5	Immersion expansion rate/%	≤2	1.33
6	Apparent relative density (g/cm^3^)	≥2.5	3.39
7	Plasticity index	≤17	8.7

**Table 2:** Chemical components of steel slag (percentage of mass).

Chemical Components	SiO_2_	CaO	MgO	Fe_2_O_3_	Al_2_O_3_	SO_3_	MnO	P_2_O_5_	Others
Concentration/%	24.35	56.34	4.11	0.2	1.63	0.23	1.04	0.002	10.62

**Table 3:** The major technical indexes of Portland cement P.O42.5.

Index			Norm	Test Result
Fineness/%			≤10	2
Initial setting time/h			≥3	4.5
Final setting time/h			6 ≤ t ≤ 10	8.5
Strength/MPa	Renitency	3 d	≥19.0	40.5
		28 d	≥42.5	58.5
	Fracture	3 d	≥3.5	5
		28 d	≥6.5	7.5

**Table 4:** The pass rates of different screen apertures (%).

Gradation Types	Sieve Size (mm)									
	19	13.2	9.5	4.75	2.36	1.18	0.6	0.3	0.15	0.075
Coarse	100	86	72	45	31	22	15	10	7	5
Intermediate	100	81	65.5	40	26.5	17.5	11.5	7.5	5	3.5
Fine	100	76	59	35	22	13	8	5	3	2
Sieving	100	82	64	41	26	16	9	7	4	2
Design	100	80	65.5	40	26.5	17.5	11.5	7	5	3

**Table 5:** Compaction test results.

Cement Content (%)	Optimal Water Content (%)	Maximum Dry Density (kg/m^3^)
4.5	4.9	2422
5	5.1	2445
5.5	5.2	2579

**Table 6:** The 7-day unconfined compressive strengths.

Cement Content	4.5%	5%	5.5%
average (MPa)	6.7	9.9	10.8
standard deviation	1.04	0.36	1.55

**Table 7:** Bending tensile strength test results.

	Destructive Load/N	Strength/MPa
average value	6659	1.99
standard deviation	290	0.09

**Table 8:** Fatigue life under different stress levels/times.

Stress Levels	0.6	0.68	0.76	0.84
average value	375,416	98,086	45,670	3673
standard deviation	47,473	15,477	11,711	1636

**Table 9:** Factor levels.

Factor Levels	$\bar{E^{*}}/GPa$	$\bar{k^{*}}$	$\bar{\sigma_{c}}/MPa$	$\bar{c}/MPa$	$\bar{\varphi }/{}^{\circ}$	$\beta_{n}/N\cdot {\left(m/s\right)}^{-1}$	μ	λ
1	2.4	0.2	34	34	10	0.2	0.2	0.2
2	3.2	0.5	29.5	29.5	25	0.4	0.5	0.5
3	4	0.8	25	25	40	0.6	0.8	0.8
4	4.8	1.1	20.5	20.5	55	0.8	1.1	1.1
5	5.6	1.4	16	16	70	1.0	1.4	1.4

**Table 10:** Orthogonal numerical test scheme and results.

Scheme	Test Factors								Test Results		
	$\bar{E^{*}}/GPa$	$\bar{k^{*}}$	$\bar{\sigma_{c}}/MPa$	$\bar{c}/MPa$	$\bar{\varphi }/{}^{\circ}$	$\beta_{n}$	μ	λ	$\sigma_{\mu }/MPa$	E/GPa	ν
1	1	1	1	4	1	1	1	2	6.860	2.731	0.227
2	1	2	2	3	5	4	5	1	5.016	1.692	0.312
3	1	3	3	2	4	2	3	5	53.298	13.474	0.118
4	1	4	5	5	3	3	2	4	20.479	8.255	0.195
5	1	5	4	1	2	5	4	3	19.025	4.808	0.266
6	2	1	2	5	4	5	5	5	48.313	16.586	0.148
7	2	2	3	1	3	1	3	4	37.581	12.247	0.100
8	2	3	5	4	2	4	2	3	13.473	6.892	0.218
9	2	4	4	3	1	2	4	2	9.422	4.104	0.351
10	2	5	1	2	5	3	1	1	2.222	1.134	0.462
11	3	1	3	3	2	3	4	1	4.412	2.557	0.490
12	3	2	5	2	1	5	1	5	37.759	19.488	0.066
13	3	3	4	5	5	1	5	4	26.431	12.138	0.142
14	3	4	1	1	4	4	3	3	25.809	8.109	0.231
15	3	5	2	4	3	2	2	2	8.826	4.261	0.396
16	4	1	5	1	5	2	4	5	49.199	31.087	0.044
17	4	2	4	4	4	3	1	4	24.978	15.507	0.105
18	4	3	1	3	3	5	5	3	24.152	9.645	0.181
19	4	4	2	2	2	1	3	2	11.626	5.935	0.357
20	4	5	3	5	1	4	2	1	2.822	2.195	0.422
21	5	1	4	2	3	4	5	2	10.579	8.325	0.142
22	5	2	1	5	2	2	3	1	3.899	2.793	0.328
23	5	3	2	1	1	3	2	5	58.939	22.603	0.099
24	5	4	3	4	5	5	4	4	32.203	14.866	0.156
25	5	5	5	3	4	1	1	3	12.948	8.576	0.270
26	1	1	2	1	2	4	1	4	31.785	9.707	0.029
27	1	2	3	4	1	2	5	3	17.119	6.002	0.167
28	1	3	5	3	5	3	3	2	7.291	3.176	0.358
29	1	4	4	2	4	5	2	1	2.835	1.298	0.395
30	1	5	1	5	3	1	4	5	44.519	11.246	0.169
31	2	1	3	2	5	1	2	3	19.738	8.661	0.082
32	2	2	5	5	4	4	4	2	6.970	4.027	0.267
33	2	3	4	1	3	2	1	1	2.401	1.588	0.424
34	2	4	1	4	2	3	5	5	55.711	14.090	0.134
35	2	5	2	3	1	5	3	4	36.430	9.214	0.197
36	3	1	5	4	3	5	3	1	3.098	2.422	0.494
37	3	2	4	3	2	1	2	5	41.352	18.157	0.060
38	3	3	1	2	1	4	4	4	40.857	12.428	0.132
39	3	4	2	5	5	2	1	3	15.941	6.064	0.272
40	3	5	3	1	4	3	5	2	11.339	4.944	0.367
41	4	1	4	5	1	3	3	3	12.538	9.078	0.302
42	4	2	1	1	5	5	2	2	12.164	5.334	0.292
43	4	3	2	4	4	1	4	1	4.226	2.603	0.379
44	4	4	3	3	3	4	1	5	51.392	18.123	0.145
45	4	5	5	2	2	2	5	4	26.253	12.702	0.181
46	5	1	1	3	4	2	2	4	35.632	17.567	0.036
47	5	2	2	2	3	3	4	3	22.889	12.473	0.16
48	5	3	3	5	2	5	1	2	6.200	5.307	0.358
49	5	4	5	1	1	1	5	1	4.149	3.529	0.404
50	5	5	4	4	5	4	3	5	43.385	19.038	0.151

**Table 11:** Statistics Fj value of the multi-factor analysis of variance.

Test Index	Test Factors							
	$\bar{E^{*}}$	$\bar{k^{*}}$	$\bar{\sigma_{c}}$	$\bar{c}$	$\bar{\varphi }$	$\beta_{n}$	μ	λ
E	11.877	3.369	0.785	2.234	1.303	0.658	0.828	97.925
$\sigma_{\mu }$	1.326	2.046	12.909	4.309	0.622	0.826	4.011	415.871
ν	1.586	5.168	0.808	1.826	1.121	0.788	0.584	81.846

**Table 12:** Influence degree of different factors on the test indexes.

Test Index	Influence Degree: High → Low							
$\sigma_{\mu }$	λ	$\bar{\sigma_{c}}$	$\bar{c}$	μ	$\bar{k^{*}}$	$\bar{E^{*}}$	$\beta_{n}$	$\bar{\varphi }$
E	λ	$\bar{E^{*}}$	$\bar{k^{*}}$	$\bar{c}$	$\bar{\varphi }$	μ	$\bar{\sigma_{c}}$	$\beta_{n}$
ν	λ	$\bar{k^{*}}$	$\bar{c}$	$\bar{E^{*}}$	$\bar{\varphi }$	$\bar{\sigma_{c}}$	$\beta_{n}$	μ

**Table 13:** Steel slag coarse aggregate contact modeling parameter values.

Number of Freeze–Thaw Cycles	Parameter Category					
	$\bar{E^{*}}/GPa$	$\bar{k^{*}}$	$\bar{\sigma_{c}}/MPa$	$\bar{c}/MPa$	$\bar{\varphi }/{}^{\circ}$	λ
0	3.2	1.0	34	34	58	0.77
5	2.8	1.0	30	28	49	0.67
10	2.6	1.0	29	25	35	0.62
20	2.2	1.0	24	23	35	0.50
30	1.8	1.0	23	23	34	0.46
40	1.4	1.0	21	23	30	0.40
50	1.4	1.0	20	22	30	0.37

**Table 14:** Contact parameters between coarse and fine aggregates of steel slag.

Number of Freeze–Thaw Cycles	Parameter Category					
	$\bar{E^{*}}/GPa$	$\bar{k^{*}}$	$\bar{\sigma_{c}}/MPa$	$\bar{c}/MPa$	$\bar{\varphi }/{}^{\circ}$	λ
0	4.0	1.0	36	34	55	0.86
5	3.4	1.0	36	31	54	0.74
10	2.8	1.0	32	31	46	0.65
20	2.6	1.0	28	29	40	0.55
30	2.3	1.0	27	28	36	0.51
40	1.9	1.0	26	26	35	0.49
50	1.8	1.0	24	24	34	0.41

**Table 15:** Steel slag fine aggregate contact modeling parameter values.

Number of Freeze–Thaw Cycles	Parameter Category					
	$\bar{E^{*}}/GPa$	$\bar{k^{*}}$	$\bar{\sigma_{c}}/MPa$	$\bar{c}/MPa$	$\bar{\varphi }/{}^{\circ}$	λ
0	2.4	1.0	30	35	58	0.64
5	2.1	1.0	29	34	56	0.58
10	1.9	1.0	27	34	48	0.53
20	1.4	1.0	24	30	39	0.45
30	1.2	1.0	23	29	37	0.42
40	0.8	1.0	23	29	36	0.39
50	0.8	1.0	22	27	36	0.36

**Table 16:** Comparison between numerical simulation of bending strength and laboratory test results.

Test Method	Laboratory Test	Simulation Test
failure load (N)	6659	6576
bend intensity (MPa)	1.99	1.97

**Table 17:** The number of freeze–thaw cycles and the bending strength of specimens.

Freeze–Thaw Cycles	0	5	10	20	30	40	50
failure load (KN)	6659	6263	5770	4582	4243	3652	3542
bend intensity (MPa)	1.99	1.88	1.73	1.37	1.27	1.09	1.06

**Table 18:** Simulated fatigue life of specimens.

Stress Level	0.6	0.68	0.76	0.84
fatigue life (times)	323825	86809	40684	3338

**Table 19:** Fatigue life under different freeze–thaw cycle stress ratios (times).

Number of Freeze–Thaw Cycles	Stress Ratio			
	0.6	0.68	0.76	0.84
0	323,825	86,809	40,684	3338
5	303,646	77,938	35,808	2799
10	278,759	71,946	30,871	2098
20	226,268	58,921	23,902	1270
30	164,722	36,354	11,731	696
40	115,890	26,134	7643	463
50	93,516	20,034	4958	283

**Table 20:** Simulation unit setting instructions.

Type of Analogue Unit	Coupling Mode of Action in the Unit
Light load fatigue	Fatigue loading 15,690 times
Light load coupling	Fatigue loading 15,690 times and freeze–thaw cycle 10 times
Heavy load fatigue	Fatigue loading 21,590 times
Heavy load coupling	Fatigue loading 21,590 times and freeze–thaw cycle 10 times

**Table 21:** Changes in bending modulus of different damage combinations.

Simulated Action Unit (Year)	Light Load Fatigue (MPa)	Light Load Coupling (MPa)	Heavy Load Fatigue (MPa)	Heavy Load Coupling (MPa)
0	8311	8311	8311	8311
1	7887	5275	7792	4886
2	7413	4262	7161	3446
3	7090	3934	6580	2945
4	6912	3684	6437	2751
5	6500	3316	6265	2589

**Table 22:** Regression analysis results.

Operating Mode	Regression Equation	$R^{2}$
Light load fatigue	$y=3095.32e^{-0.2553x}+3184.01$	0.9907
Light load coupling	$y=4226.04e^{-0.779x}+2079.26$	0.9857
Heavy load fatigue	$y=3671.91e^{-0.2987x}+2649.96$	0.9885
Heavy load coupling	$y=5545.36e^{-0.7213x}+827.08$	0.9879

## Data Availability

Some or all data, models, or codes that support the findings of this study are available from the corresponding author upon reasonable request, including the specific dimensional stiffness.
